# Serial Block-Face Scanning Electron Microscopy Reveals That Intercellular Nuclear Migration Occurs in Most Normal Tobacco Male Meiocytes

**DOI:** 10.3389/fpls.2021.672642

**Published:** 2021-05-07

**Authors:** Sergey Mursalimov, Nobuhiko Ohno, Mami Matsumoto, Sergey Bayborodin, Elena Deineko

**Affiliations:** ^1^Laboratory of Plant Bioengineering, Institute of Cytology and Genetics, Siberian Branch of Russian Academy of Sciences, Novosibirsk, Russia; ^2^Department of Anatomy, Division of Histology and Cell Biology, School of Medicine, Jichi Medical University, Shimotsuke, Japan; ^3^Division of Ultrastructural Research, National Institute for Physiological Sciences, Okazaki, Japan; ^4^Section of Electron Microscopy, Supportive Center for Brain Research, National Institute for Physiological Sciences, Okazaki, Japan

**Keywords:** volume electron microscopy (vEM), cytomixis, male meiosis, *Nicotiana*, serial block-face scanning electron microscopy, nuclear migration, intercellular channels

## Abstract

Serial block-face scanning electron microscopy (SBF-SEM) was used here to study tobacco male meiosis. Three-dimensional ultrastructural analyses revealed that intercellular nuclear migration (INM) occurs in 90–100% of tobacco meiocytes. At the very beginning of meiosis, every meiocyte connected with neighboring cells by more than 100 channels was capable of INM. At leptotene and zygotene, the nucleus in most tobacco meiocytes approached the cell wall and formed nuclear protuberances (NPs) that crossed the cell wall through the channels and extended into the cytoplasm of a neighboring cell. The separation of NPs from the migrating nuclei and micronuclei formation were not observed. In some cases, the NPs and nuclei of neighboring cells appeared apposed to each other, and the gap between their nuclear membranes became invisible. At pachytene, NPs retracted into their own cells. After that, the INM stopped. We consider INM a normal part of tobacco meiosis, but the reason for such behavior of nuclei is unclear. The results obtained by SBF-SEM suggest that there are still many unexplored features of plant meiosis hidden by limitations of common types of microscopy and that SBF-SEM can turn over a new leaf in plant meiosis research.

## Introduction

Intercellular nuclear migration (INM), also called cytomixis, is an enigmatic phenomenon that can be seen in plant male meiocytes. In this process, nuclei migrate between cells through special intercellular channels called cytomictic channels (CCs), which are considerably larger than plasmodesmata (Mursalimov et al., 2010). This unique phenomenon was discovered over a century ago in gymnosperms (Arnoldy, 1900) and then was observed in angiosperms during male meiosis of *Crocus vernus* (Koernicke, 1901). The term *cytomixis* was proposed by Gates (1908, 1911), who studied this phenomenon in meiocytes of *Oenothera* sp. The development of electron microscopy techniques made a significant contribution to INM investigation. By these techniques, CCs were discovered, which enable the migration of such big organelles like nuclei (Heslop-Harrison, 1966). It was also shown that not only chromatin or chromosomes alone migrate through the CCs, but so does the nucleus enclosed within the nuclear membrane (Feijó and Pais, 1989). Analysis of post-translational histone modifications in the migrating chromatin as well as TUNEL and comet assays revealed that the migrating chromatin and cells involved in INM have no detectable markers of inactivation or damage (Mursalimov and Deineko, 2018). INM during male meiosis has been noted in hundreds of angiosperms and in some quillwort species (He et al., 2004; Pécrix et al., 2011; Mursalimov et al., 2013). CCs are seen in male meiocytes of ferns and horsetails; however, INM *per se* has not been detected in these species (Lehmann et al., 1984; Gabarayeva et al., 2011). In most cases, INM can be observed in zygotene and pachytene with highly varied frequency, and it is generally accepted that INM results in the formation of micronuclei (Barton et al., 2014; Reis et al., 2016).

Despite the high prevalence of INM in plant male meiosis, its causes, mechanisms, and consequences are still unclear. On the one hand, INM is regarded by most researchers as a meiotic deviation that does not deserve close attention. Thus, this phenomenon is simply ignored in many works on plant meiosis. On the other hand, some researchers consider INM a process that leads to aneuploid- or unreduced-pollen formation (Farooq et al., 2014; Fakhri et al., 2016; Djafri-Bouallag et al., 2019). This point of view is not supported by sufficient experimental evidence. Attempts to study INM by common cytological techniques have come to a standstill. One of the main obstacles to INM research is inaccessibility of intact male meiocytes for direct analysis because they are hidden inside an anther surrounded by layers of nourishing and protective cells. Common cytological techniques like visualization of cells on squashed preparation and tissue sectioning combined with light microscopy (LM) or ultrathin sectioning combined with transmission electron microscopy (TEM) provide only fragmentary information about INM. The mechanical impact on meiocytes on squashed preparations changes their structure and disrupts intercellular channels. In comparison, all kinds of sectioning preserve meiocyte structure but provide only two-dimensional data, and all the information outside a section plane is lost.

Thus, there is a need for new approaches to the study of INM in plant meiosis, and three dimensional visualization using serial block-face scanning electron microscopy (SBF-SEM) is one method of approach. SBF-SEM is a relatively new technique that allows for serial sectioning and imaging of resin-embedded material with subsequent digital alignment of hundreds and thousands of ultrastructural pictures and three-dimensional tissue reconstruction. This method was developed to study nervous tissues (Denk and Horstmann, 2004), and now it is successfully used for plant cell analysis (Kittelmann et al., 2016; Płachno et al., 2017; Arcalís et al., 2020); (Boulogne et al., 2020). SBF-SEM has never been employed to study plant meiosis. Nonetheless, it appears that SBF-SEM is an excellent way to investigate the real picture of such processes as INM that (i) are normally hidden inside organs, (ii) can be easily influenced by a mechanical impact, and (iii) need to be researched at high resolution.

In this study, we utilized SBF-SEM to investigate INM in tobacco male meiosis. The INM was analyzed in samples where cells were neither mechanically damaged nor lost. For the first time, the exact number of the meiocytes involved in INM was determined, and the number of CCs connecting meiocytes was estimated. We found that 90–100% of meiocytes in a tobacco anther are involved in INM as a nuclear donor, recipient, or both. Nucleus–nucleus contacts between the migrating nuclei and the nuclei of neighboring meiocytes were observed. No micronuclei were found in the studied cells. The successful observation of unusual behavior of nuclei in tobacco meiocytes—that has been hidden from researchers by the LM and TEM limitations for decades—means that SBF-SEM can open a whole new chapter in plant meiosis research.

## Materials and Methods

### Plant Growth and Sampling

A wild-type tobacco line (*Nicotiana tabacum* L. cv. Petit Havana SR1) with high pollen fertility was used in this work. The plants were grown in a greenhouse with a photoperiod of 16/8 h (day/night) at a temperature of 22/18°C (day/night). One anther per plant (14 total) was randomly chosen for analysis. One anther from every flower bud subjected to fixation was analyzed under a light microscope to estimate the meiotic stage.

### Observations and Image Analyses by SBF-SEM

Anthers were fixed with 2.5% paraformaldehyde and 2.5% glutaraldehyde in 0.08 M cacodylate buffer (pH 7.2) on ice overnight. After that, the tissue was washed three times for 15 min with 1 × phosphate-buffered saline (PBS, pH 7.2). Next, the anthers were mounted in 3% low-melting-point agarose in 1 × PBS and cut on a microtome with a vibrating blade (HM 650V; Microm, Germany) into 200 μm pieces. Then, they were postfixed with a mixture of 1.5% potassium ferrocyanide and 2% osmium tetroxide in 1 × PBS for 1 h at 4°C. The samples were next washed four times for 3 min in MilliQ water at room temperature and incubated in a 1% solution of thiocarbohydrazide (Sigma-Aldrich, USA) for 20 min at 60°C. After that, the samples were washed four times for 3 min in MilliQ water and placed in 2% aqueous osmium tetroxide for 30 min incubation, then the tissue was washed again four times for 3 min in MilliQ water and incubated overnight in 2% aqueous uranyl acetate at 4°C. The samples were then rinsed four times for 3 min in MilliQ water, incubated in a freshly prepared Walton's lead aspartate for 2 h at 50°C, washed four times for 3 min in MilliQ water, and dehydrated in 30, 50, 70, and 96% ethanol solutions for 10 min each, one time in 4°C 96% ethanol for 10 min, and then three times for 20 min in acetone at room temperature. After the dehydration, the samples were placed in a mixture of Araldite (Fluka, Switzerland) epoxy resin and acetone in ratios 1:2, 1:1, and 2:1 in that order, with incubation for 1 h at each step at room temperature. Then, the samples were kept in 100% resin overnight at room temperature and polymerized over two nights at 60°C.

The polymerized samples were cut out and glued with silver-containing conductive paste (KAKEN TECH, Japan) onto aluminum specimen pins and evaporated with gold in vacuum. Serial images were acquired with a ΣIGMA field emission scanning electron microscope (Carl Zeiss, Germany) equipped with 3View, a system with a built-in ultramicrotome and a back-scattered electron detector (Gatan, USA), at a resolution of 6.3 nm/pixel in X and Y directions and 40 nm in the Z direction. The size of the imaged area was 51.6096 × 51.6096 μm, which resulted in an image resolution of 8192 × 8192 pixels. From 387 to 3,689 serial images were obtained from every sample. The images from each dataset were processed in the Fiji software (Schindelin et al., 2012). Manual image segmentation was performed with Microscopy Image Browser (Belevich et al., 2016). Every image was analyzed, then nuclei and cell walls were labeled using a pen stylus and touch screen. Three-dimensional tissue reconstruction and its analyses were performed in Amira 6.2.0 software (FEI Visualization Science Group, Hillsboro, OR, USA).

## Results

### Most of Tobacco Male Meiocytes Participate in INM

**Figure 1:**
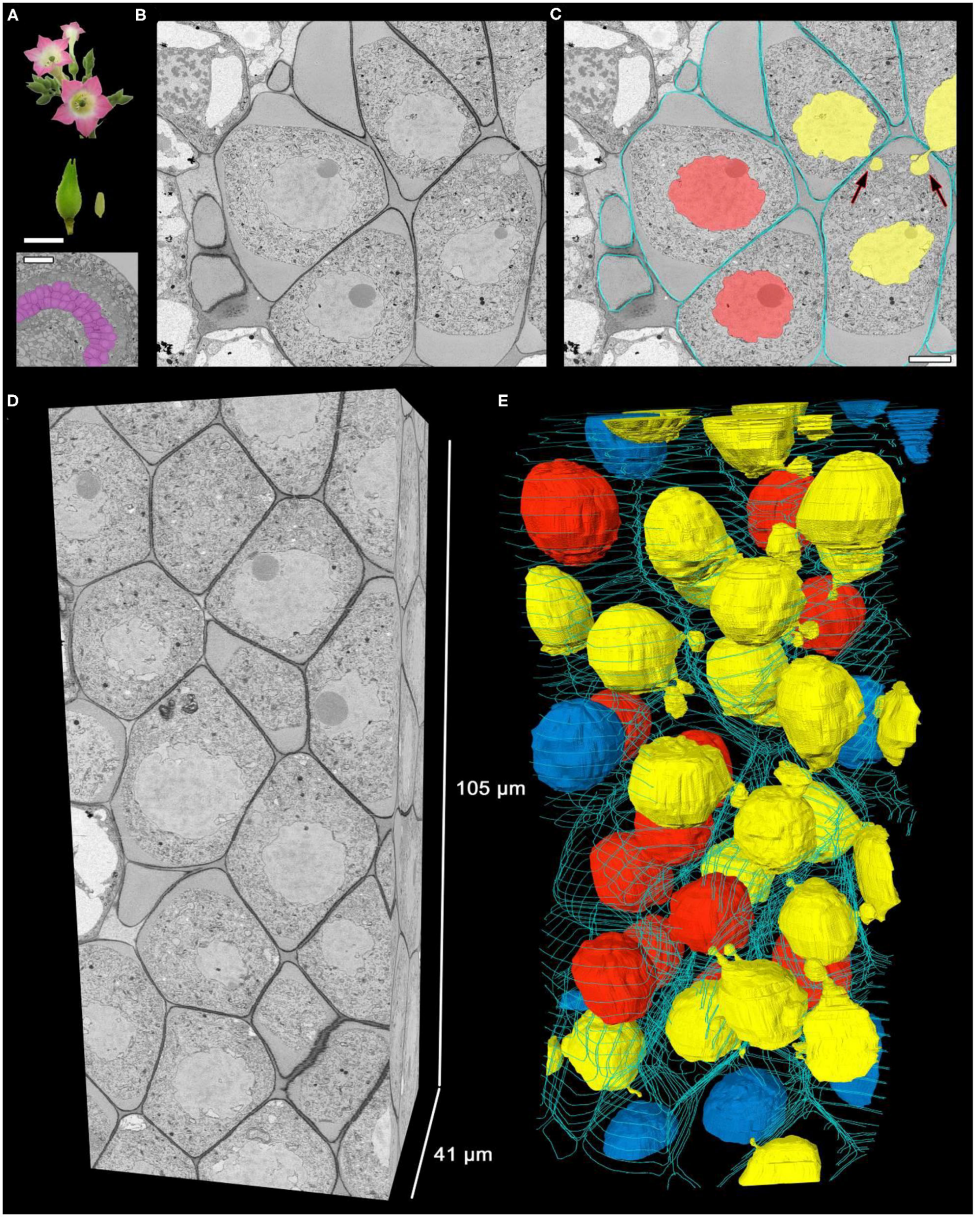
Three-dimensional reconstruction of male meiocytes in a tobacco anther. **(A)** Tobacco flowers, a flower bud, an anther, and transverse section of an anther locule. The purple color indicates meiocytes. **(B,C)** A meiocyte micrograph obtained by SBF-SEM at pachytene. An original image **(B)** and the same image after cell walls and nuclei were labeled **(C)**. **(D,E)** Three-dimensional reconstructions of a scanned tissue fragment from 2,626 serial images. The whole unlabeled tissue fragment **(D)** and labeled nuclei and cell walls **(E)**. Yellow denotes migrating nuclei, red non-migrating nuclei, blue partially scanned nuclei (their status is not clear), and turquoise the outer layer of cell walls, labeled on every 50th slice. The arrows point to the NPs crossing a cell wall. Scale bars are 5 mm and 50 μm in **(A)** and 5 μm in **(C)**.

Fourteen anthers, each from an individual tobacco plant, were randomly collected for analysis (Figure 1A). A random tissue fragment from every anther containing meiocytes was analyzed by SBF-SEM. As a result, serial ultrastructural images of tobacco meiocytes were obtained for every sample (Figure 1B). Then, using software, meiocyte nuclei and cell walls were labeled in these images (Figure 1C), and three-dimensional structure of the tissue fragments was reconstructed (Figures 1D,E; Supplementary Video 1).

In a typical tobacco meiocyte involved in INM, a nucleus approached the cell wall and formed nuclear protuberances (NPs) that crossed the cell wall through one or a few CCs and extended into the cytoplasm of a neighboring cell. Thus, some meiocytes became nuclear donors (Figures 1C,E, yellow nuclei). The nuclei of the other meiocytes did not move (Figures 1C,E, red nuclei), but these cells could still take part in the INM as recipients of the migrating nuclei. One meiocyte could be both a nuclear donor and recipient at the same time. A nucleus from one donor cell could migrate simultaneously into two recipient cells as well as two nuclei could migrate into one recipient cell (Figures 1C,E; Supplementary Video 1).

**Figure 2:**
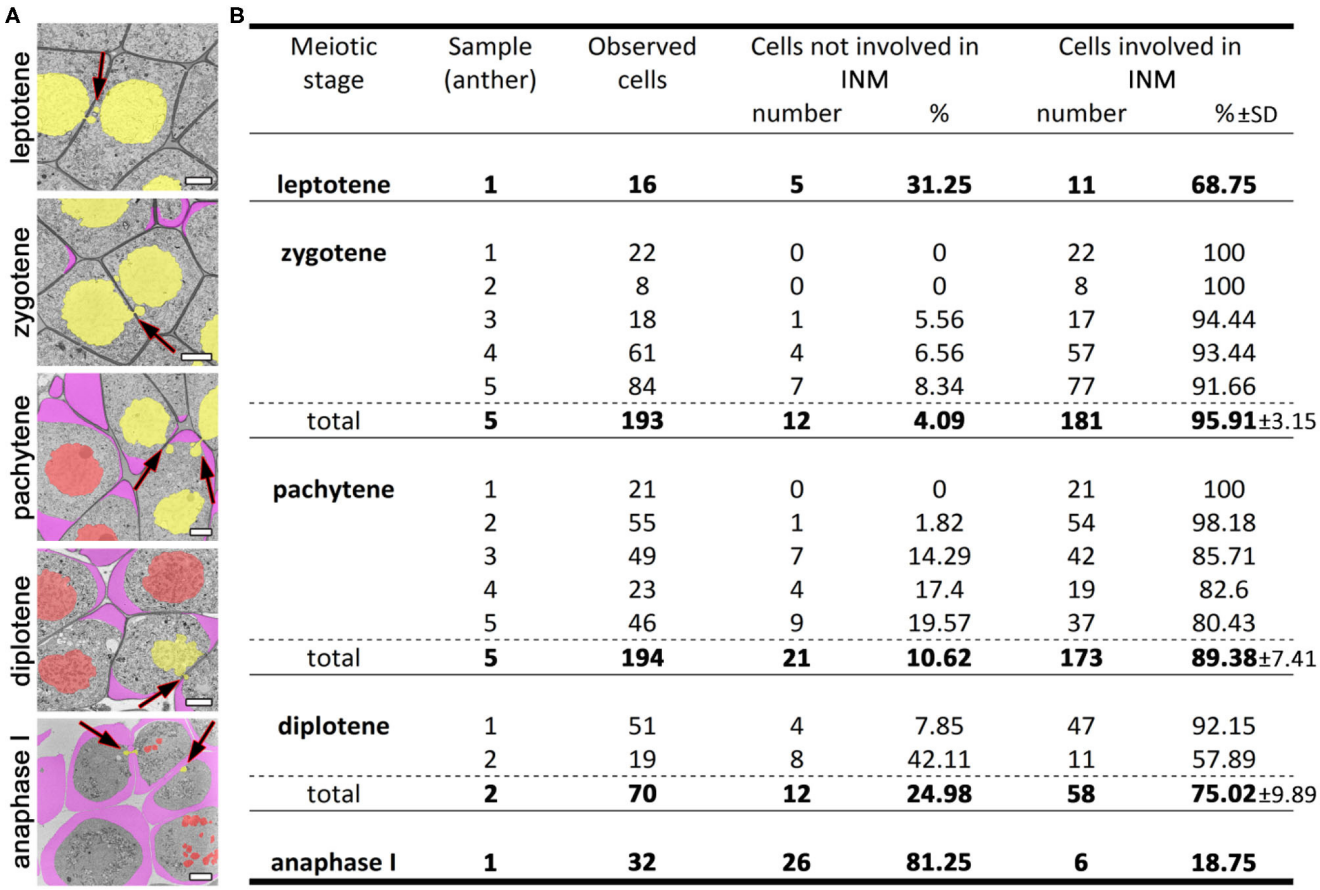
INM frequencies at different meiotic stages. **(A)** Tobacco meiocytes from leptotene to anaphase I. Yellow denotes migrating nuclei, red non-migrating nuclei, and purple a callose wall; the arrows indicate the NPs crossing a cell wall. **(B)** Statistical data on the INM frequencies. SD, standard deviation. Scale bars are 5 μm.

We studied tobacco meiocytes at leptotene, zygotene, pachytene, and diplotene of prophase I and at anaphase I (Figure 2A; Supplementary Figures 1–5). Special attention was given to zygotene and pachytene. A total of 505 tobacco meiocytes were analyzed, 429 of them were involved in INM and 76 were not (Figure 2B). The observed INM frequency was quite surprising: nearly 100% of meiocytes were involved in the INM as a donor, recipient, or both at certain stages. At leptotene, ~70% of the tobacco meiocytes participated in the INM. At zygotene, INM frequency increased up to 90–100% in every tobacco anther studied at this stage. At pachytene, it was ~80–100%, and the INM frequency gradually decreased throughout meiotic stages. At anaphase I, chromatin inside CCs was noted in fewer than 20% of meiocytes. We were unable to find any samples without the INM in the studied material. Besides the high frequency of the INM, we did not find any other visible deviations in the analyzed meiocytes.

### The Separation of NPs From the Migrating Nuclei Is Not Detectable

**Figure 3:**
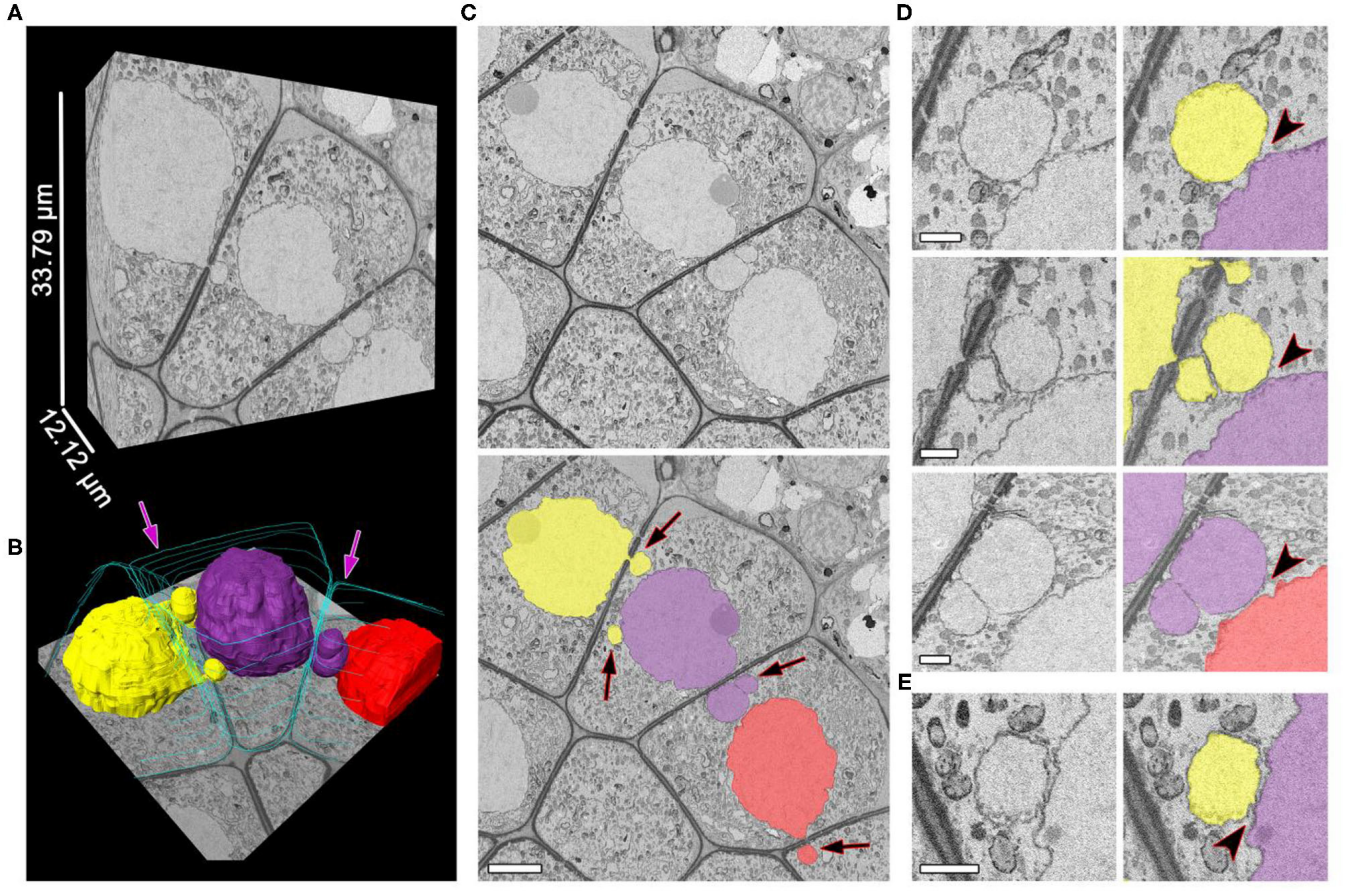
Unidirectional migration of nuclei between meiocytes at zygotene. **(A,B)** Three-dimensional reconstructions of the scanned tissue fragment by means of 303 serial images. The whole unlabeled tissue fragment **(A)** and labeled nuclei and cell walls **(B)**. **(C)** The original and labeled micrograph. **(D,E)** Nucleus–nucleus contacts between NPs and nuclei of the neighboring cells, enlarged. The space between nuclear membranes is visible in **(D)** and not visible in **(E)**. Yellow, purple, and red denote individual nuclei (all of them are involved in the INM), and turquoise indicates cell walls, labeled on every 50th slice; the arrows indicate the NPs crossing a cell wall; the arrowheads point to nucleus–nucleus contact areas. Scale bars are 5 μm in **(B)** and 1 μm in **(D,E)**.

**Figure 4:**
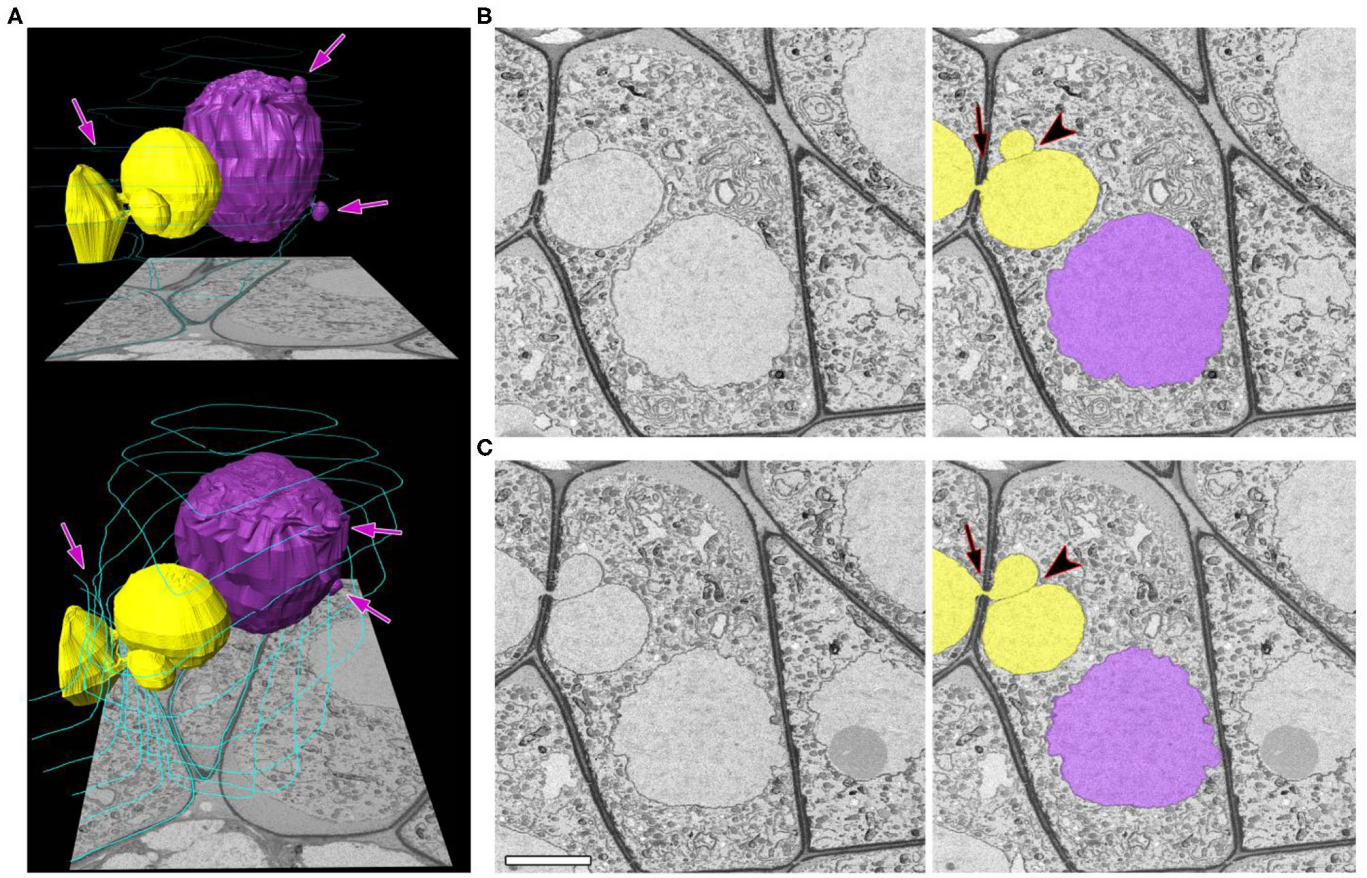
A zygotene meiocyte that has a potential to become binucleated. **(A)** Three-dimensional reconstructions from 464 serial images of labeled nuclei and cell walls in two views at different angles. **(B,C)** Original and labeled micrographs with the biggest size of the NPs. Yellow and purple denote individual nuclei (both are involved in the INM), and turquoise represents cell walls, labeled on every 50th slice. The arrows indicate the NPs crossing a cell wall, and the arrowheads point to contact areas between the NPs. The scale bar is 5 μm.

The separation of NPs from the migrating nuclei followed by micronuclei formation were not detectable by SBF-SEM in tobacco meiocytes. We did not find binucleated meiocytes either. All the observed NPs after crossing the cell wall were found to be connected with the nucleus of the donor cell. At zygotene, ~70% of NPs approached nuclei of recipient cells (Figures 3A–C; Supplementary Video 2). Their nuclear membranes got very close at this stage (Figure 3D, arrowheads), and the gap between membranes of two nuclei became indistinguishable in some cases (Figure 3E, arrowhead). As a rule, NPs formed by the migrating nuclei were oriented in the same direction. Thus, meiocytes often formed chainlike structures connected by NPs (Figures 3A–C; Supplementary Video 2). In most cells, the size of NPs entering the cytoplasm of neighboring cells was ~3–5% of the migrating-nucleus volume. Nevertheless, in one meiocyte, we observed two NPs with the size of ~40% of its nucleus (Figure 4; Supplementary Video 3). These NPs migrated through two separate CCs but were still located very closely without visible space between their nuclear membranes (Figures 4B,C, arrowheads).

### Fewer Than 15% of CCs Are Involved in the INM

**Figure 5:**
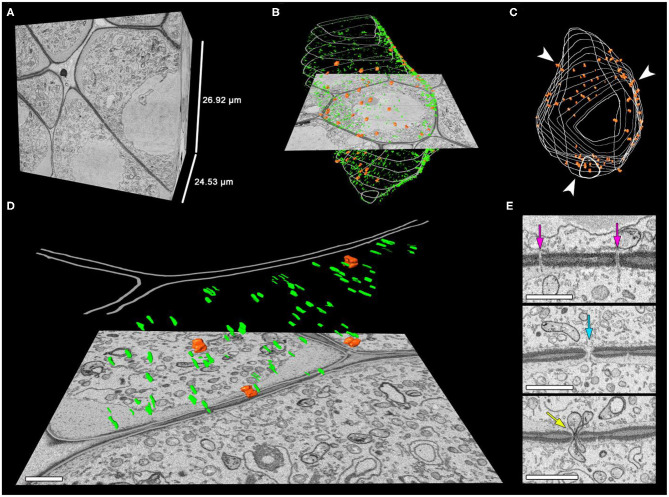
Distribution of the intercellular channels in the tobacco meiocyte cell wall. **(A–C)** Three-dimensional reconstructions of the scanned tissue fragment from 673 serial images. The whole unlabeled tissue fragment that contains one meiocyte **(A)**, labeled CCs, plasmodesmata, and cell wall **(B)**, with only CCs and the cell wall labeled in the z-projection. **(D)** An enlarged cell wall fragment. **(E)** Sections with plasmodesmata and CCs. Orange represents CCs, green plasmodesmata, and gray the cell wall. Arrowheads indicate the CC clustering regions, the purple arrow points to plasmodesmata on a section, the turquoise arrow indicates a CC on a section filled with cytoplasm, and the yellow arrow points to a CC on a section with two plastids inside. Scale bars are 1 μm.

After three-dimensional reconstruction of individual meiocytes' structures (Figures 5A–C; Supplementary Video 4), we observed two types of intercellular channels in their cell wall: CCs and plasmodesmata. These types of channels could be identified easily. Plasmodesmata were found to be distributed uniformly in the meiocyte cell wall (Figures 5B,D). CCs formed clusters in a few cell regions (Figures 5B,C). Unlike CCs, plasmodesmata had distinct internal structure (Figure 5E). CCs were more like holes in the cell wall filled with cytoplasm, migrating nuclei, or other organelles such as vesicles or plastids (Figure 5E; Supplementary Video 5).

For the first time, we were able to determine the exact number of CCs that connect tobacco meiocytes (Table 1). At the very beginning of meiosis (leptotene), an average meiocyte had ~140 CCs in its cell wall. At the following prophase I stages, the CC number decreased quite fast, and at pachytene, an average meiocyte had ~25 channels. After that, the CC number did not change significantly at least until anaphase I, the last stage we checked. The number of CCs per cell was variable, but their dynamics were obvious in all samples; the highest number of CCs connected tobacco meiocytes at leptotene and then decreased due to callose wall formation (Figure 2A).

**Table 1:** The number of CCs in tobacco meiocytes at different meiotic stages.

**Meiotic stage (three meiocytes were chosen randomly for every stage)**	**Number of CCs per cell (standard deviation)**	**CCs involved in nuclear migration**	
		**Number (standard deviation)**	**%**
Leptotene	143.33 (18.01)	7 (3.51)	4.88
Zygotene	90.66 (16.99)	6.33 (0.81)	6.98
Pachytene	25.33 (7.68)	3.66 (2.38)	14.44
Diplotene	21.66 (18.52)	0.66 (4.52)	3.04
Anaphase I	22.33 (9.66)	0.33 (4.72)	1.47

Even though tobacco meiocytes contained dozens of CCs, few of them were engaged in the INM. At zygotene and pachytene, when INM frequency was the highest, three to seven CCs had NPs inside in a meiocyte, i.e., fewer than 15% of CCs took part in the INM (Table 1). This number includes both NPs formed by the cell itself and NPs that the cell received from neighboring cells. We observed the formation of CCs only between meiocytes. CCs connecting meiocytes and tapetum cells were not detected.

The plasmodesmata showed dynamics similar to those of CCs during meiosis. Their number was the highest at the leptotene stage and then gradually decreased. Unlike CCs, there were no plasmodesmata left in the meiocyte cell wall at anaphase I.

## Discussion

### INM in Tobacco Meiosis Has Been Greatly Underestimated

A common laboratory tobacco line was used in this work. Previously, we studied INM in this line by different approaches using various types of fixatives, staining protocols, and types of microscopy (Mursalimov and Deineko, 2018). In general, the cytological picture of the INM (and its dynamics throughout meiotic stages) has been the same regardless of what type of treatment protocol and what type of microscopy are used for the analysis. Nevertheless, the high INM frequency has always stayed hidden. Using squashed preparations combined with LM, we estimated the INM frequency in plants of this line at 0.6% (Mursalimov and Deineko, 2018). In the present study, SBF-SEM revealed that in fact, the INM frequency in their meiosis is ~90–100%, not 0.6%. Thus, by SBF-SEM, we found that in tobacco meiosis, INM frequency is ~150-fold higher than estimated before by LM. We believe that there are two reasons for such a big discrepancy. The first reason is the mechanical impact on cells on squashed preparations that disrupts NPs and prevents their counting. The second reason is the limits of LM resolution: some NPs are too small to be detected this way.

It is important to note that INM is not a unique feature of this line or tobacco in general. INM in male meiosis has been observed in many plant species (He et al., 2004; Pécrix et al., 2011; Mursalimov et al., 2013). The INM frequency estimated by means of squashed preparations combined with LM is highly varied among species and even within a species if this parameter is estimated by different authors. It can vary from <1 to 80–100% of male meiocytes in an anther (Li et al., 2009; Reis et al., 2016; Kaur and Singhal, 2019). Such high variation of the numbers makes them doubtful. In light of our results obtained by SBF-SEM, it has to be admitted that squashed preparations combined with LM are not suitable for correct estimation of INM frequency. In this field, until male meiosis of other plant species is analyzed by SBF-SEM or similar methods, it will be impossible to say with confidence that the high INM frequency is a unique feature of tobacco or is also present in other species.

### The Micronuclei Formation Is Not the Main Consequence of INM in Tobacco

It is generally accepted that micronucleus formation is the main consequence of INM in all the plant species where INM has been detected including tobacco (Mursalimov and Deineko, 2011; Barton et al., 2014; Reis et al., 2016). Here, SBF-SEM revealed that for tobacco, this point of view is incorrect. In this work, we did not find a single case of micronuclei formation after INM in tobacco meiosis. Misinterpretation of the LM and TEM results on squashed preparations and sections may explain the previous incorrect conclusions about the INM consequences in tobacco and possibly in other species. As mentioned above, these techniques' limitations lead to the underestimation of INM frequency, and apparently for the same reasons, micronucleuslike structures are seen. Many of NPs inside CCs can be disrupted on squashed preparations. Even if some of them persist, they are often too small to be detected by LM. When INM is studied by TEM, NPs inside CCs may be invisible outside the plane of an ultrathin section. By contrast, NPs that already left CCs and entered the cytoplasm of a recipient cell are more prominent due to their bigger size. This set of NPs cannot hide from analysis so easily. This state of affairs may lead to the observation of the micronucleuslike structures. Here, in all the observed cases, NPs inside CCs were not disrupted, and the NPs located in the cytoplasm of recipient cells did not lose the connection with the main part of their nucleus staying in a donor cell.

There are three other possible scenarios for the migrating nuclei if they do not produce micronuclei: (i) a whole nucleus can cross the cell wall without dividing into parts and form a binucleated cell, (ii) NPs can fuse with a nucleus of a recipient cell and become undetectable, and (iii) NPs can return into the donor cell without any consequences. The formation of binucleated meiocytes as a result of INM has been described in several plant species. It has been shown that a whole nucleus can migrate from a donor to recipient cell, thereby forming one binucleated and one empty meiocyte. Presumably, such binucleated meiocytes can produce unreduced pollen (Singhal and Kumar, 2008; Tsvetova and Elkonin, 2013; Sidorchuk et al., 2016). The frequency of binucleated-meiocyte formation as a consequence of INM has not been estimated before for any plant species. In our study, SBF-SEM revealed that in tobacco meiosis, one in 429 meiocytes involved in INM has a potential to become a binucleated cell. Thus, due to its low frequency, the binucleated-meiocyte formation cannot be the main consequence of the INM in tobacco.

The nucleus–nucleus contacts observed by SBF-SEM during the INM suggest that nuclear fusion is a possible consequence of this process. Nuclear fusions during INM in tobacco male meiocytes have already been documented by TEM. After the fusion, two nuclei stay in their own cells but make contact through CCs and share one nuclear membrane forming a bridgelike structure filled with chromatin (Mursalimov and Deineko, 2011). Nonetheless, only a few cases of such bridgelike-structure formation have been registered by TEM, and they have never been detected by LM in tobacco or by LM/TEM in other species. Here, SBF-SEM indicated that the migrating nuclei in tobacco meiocytes may fuse under certain conditions. Nonetheless, we did not directly see nuclear fusions by means of SBF-SEM. It can be hypothesized that distinct bridgelike structures between tobacco nuclei either form with low frequency or exist for a very short period, and that is why it is difficult to detect them.

It has been suggested many times that INM can cause aneuploid- or unreduced-pollen formation (Farooq et al., 2014; Fakhri et al., 2016; Djafri-Bouallag et al., 2019). In this context, nucleus–nucleus contacts observed in tobacco meiocytes look quite intriguing, and it can be assumed that they result in changes of the chromatin amount in nuclei of both donor and recipient cells. The nuclei can gain or lose some chromatin/chromosomes. As a result, pollen with altered karyotype will be formed. It is well-known that polyploidization is one of the major driving forces behind plant evolution, and the unreduced- or aneuploid-pollen formation plays a key role in this process (De Storme and Mason, 2014). In contrast to animals and yeast, plant meiosis is a rather robust process that has no critical checkpoints (Wijnker and Schnittger, 2013). Accordingly, plant meiotic division continues even if the chromosome number changes or their homologous pairing is absent. In this context, INM can be an additional way to increase genetic diversity because of the formation of aneuploid or unreduced pollen. It is noteworthy that INM is seen in plant meiocytes at the same meiotic stages when crossing over occurs. This means that meiocytes are primed for genetic recombination at this moment.

Obviously, this hypothesis requires additional experimental evidence probably obtained by approaches that have not been developed yet. Such techniques as two-photon microscopy and light sheet microscopy have a potential for INM analysis in live meiocytes inside an anther but only if these methods are adapted to the tobacco model because there is still a possibility that the high INM frequency is only a tobacco feature. For now, these techniques yield impressive results in studies on the meiosis of *Agapanthus umbellatus* (Feijó and Cox, 2001), maize (Sheehan and Pawlowski, 2009), and *Arabidopsis* (Valuchova et al., 2020). The problem is that these models are not suitable for INM investigation. INM has never been detected in the meiosis of *A. umbellatus* and *Arabidopsis*. On the other hand, many years ago, INM was detected in maize by McClintock (1929) in triploid plants and later by Caetano and Pagliarini (1997) in inbred maize plants. It seems that either INM frequency is too low in normal maize meiosis or the size of NPs is too small to be detectable by LM. As far as we know, these protocols have not been adapted to tobacco anthers owing to their dense non-transparent outer cell layers.

Because of such unusual behavior of nuclei during INM, the discussion of the artificial nature of INM was started only after its discovery a century ago and continues to this day. We believe that at present, there are enough data to prove that INM is not an artifact of chemical fixation or other technical factors. INM has been studied by different groups using 18 types of fixation including cryofixation. The material fixed by different protocols has been studied on squashed preparations and tissue sections embedded in paraffin, polyethylene glycol, methacrylates, and epoxy resins. In all these studies, the general picture of the observed INM has been the same. These data are summarized in a review article (Mursalimov and Deineko, 2018). By means of *Drosophila* ring channels as an example, it was demonstrated that loosing of cytoskeletal anchors is not the cause of INM because passively migrating nuclei cannot pass even through big intercellular channels (ring channels are 15-fold larger than the CCs) and only plug them (Ogienko et al., 2008). Thus, INM is supposed to be an active process, where the nucleus should be actively dragged into another cell to successfully pass through intercellular channels. Some signs of the interconnection between the migrating chromatin and cytoskeleton in plant meiocytes have been documented (Barton et al., 2014; Mursalimov et al., 2019). We still know very little about the cellular machinery of INM; however, the experimental data obtained for a 100 years of INM study indicate that INM is not an artifact.

Taken together, our results lend themselves to the following preliminary interpretation of the observed phenomenon. Nuclei migrate through CCs at leptotene and zygotene. There is a chance that they can reach nuclei of neighboring cells with yet unknown consequences. At pachytene, the migrating nuclei return to their initial position in donor cells, and the meiocytes continue meiosis independently. It appears that meiocytes complete the INM before the nuclear-membrane disappearance at the end of the first meiotic prophase. The presence of chromatin inside CCs in later meiotic phases such as anaphase I is most likely a deviation from typical INM. In this case, migrating nuclei probably do not manage to return to the donor cells completely before their nuclear membrane disappears, and the chromatin gets stuck inside CCs.

In this study, SBF-SEM also revealed that CC functions are not limited to INM. In fact, only a few CCs are involved in this process in every meiocyte. In addition, there are some meiocytes in an anther that contain dozens of CCs and do not participate in INM at all. Due to the possibility of free movement of cytoplasm and small organelles through CCs, their main function may be the transfer signaling molecules and nutrients that is essential for synchronous meiotic division.

### Conclusion

Thus, SBF-SEM indicates that the INM in tobacco male meiosis has been greatly underestimated so far. Nearly 100% of meiocytes are involved in the INM at certain meiotic stages. The formation of micronuclei, a generally accepted consequence of INM, was not observed in this study. INM, which has been regarded as a meiotic deviation by many authors, in reality, seems to be a constant part of normal tobacco male meiosis; however, the reason for such behavior of nuclei is unclear. Nevertheless, it is amazing how the real picture of INM in tobacco meiosis has been hiding in plain sight for decades owing to the limitations of LM and TEM. Definitely, more researchers must try to tackle this problem, thereby starting a whole new chapter in the study of plant meiosis by SBF-SEM and other new methods.

## Data Availability Statement

The original contributions presented in the study are included in the article/Supplementary Material, further inquiries can be directed to the corresponding author.

## Author Contributions

SM designed and coordinated the study, prepared the material, interpreted the data, and drafted the manuscript. NO participated in the interpretation of the data and drafting of the manuscript. MM performed the microscopy analysis. SB participated in the material preparation and interpreted the data. ED conceived the study, interpreted the data, and revised the manuscript critically. All authors contributed to the article and approved the submitted version.

## Conflict of Interest

The authors declare that the research was conducted in the absence of any commercial or financial relationships that could be construed as a potential conflict of interest.

## Supplementary Material

Supplementary Video 1 Three-dimensional reconstruction of male meiocytes at pachytene in a tobacco anther. Reconstructed from 2,626 serial images. Yellow denotes migrating nuclei, red non-migrating nuclei, blue partially scanned nuclei (their status is not clear), and turquoise the outer layer of cell walls, labeled on every 50th slice.

Supplementary Video 2 Unidirectional migration of nuclei between meiocytes at zygotene. Reconstructed from 303 serial images. Yellow, purple, and red denote individual nuclei (all of them are involved in the INM), and turquoise indicates cell walls, labeled on every 50th slice.

Supplementary Video 3 A zygotene meiocyte that has a potential to become binucleated. Reconstructed from 464 serial images. Yellow and purple denote individual nuclei (both are involved in the INM), and turquoise represents cell walls, labeled on every 50th slice.

Supplementary Video 4 Distribution of the intercellular channels in the tobacco meiocyte cell wall. Reconstructed from 673 serial images. Orange represents CCs, green plasmodesmata, and gray the cell wall.

Supplementary Video 5 An enlarged cell wall fragment. Reconstructed from 130 serial images. Orange represents CCs, green plasmodesmata, and gray the cell wall.

Supplementary Figure 1 Tobacco meiocytes at leptotene (original unlabeled image from Figure 2A).

Supplementary Figure 2 Tobacco meiocytes at zygotene (original unlabeled image from Figure 2A).

Supplementary Figure 3 Tobacco meiocytes at pachytene (original unlabeled image from Figure 2A).

Supplementary Figure 4 Tobacco meiocytes at diplotene (original unlabeled image from Figure 2A).

Supplementary Figure 5 Tobacco meiocytes at anaphase I (original unlabeled image from Figure 2A).

## Abbreviations

SBF-SEM: serial block-face scanning electron microscopy
vEM: volume electron microscopy
TEM: transmission electron microscopy
LM: light microscopy
INM: intercellular nuclear migration
NP: nuclear protuberance
CC: cytomictic channel
TUNEL: Terminal deoxynucleotidyl transferase dUTP nick end labeling
PBS: phosphate-buffered saline.
