# Corrigendum: Serial Block-Face Scanning Electron Microscopy Reveals That Intercellular Nuclear Migration Occurs in Most Normal Tobacco Male Meiocytes

**DOI:** 10.3389/fpls.2022.847408

**Published:** 2022-01-25

**Authors:** Sergey Mursalimov, Nobuhiko Ohno, Mami Matsumoto, Sergey Bayborodin, Elena Deineko

**Affiliations:** ^1^Laboratory of Plant Bioengineering, Institute of Cytology and Genetics, Siberian Branch of Russian Academy of Sciences, Novosibirsk, Russia; ^2^Department of Anatomy, Division of Histology and Cell Biology, School of Medicine, Jichi Medical University, Shimotsuke, Japan; ^3^Division of Ultrastructural Research, National Institute for Physiological Sciences, Okazaki, Japan; ^4^Section of Electron Microscopy, Supportive Center for Brain Research, National Institute for Physiological Sciences, Okazaki, Japan

**Keywords:** volume electron microscopy (vEM), cytomixis, male meiosis, *Nicotiana*, serial block-face scanning electron microscopy, nuclear migration, intercellular channels

There is an error in the Funding statement. The correct number for the Russian Foundation for Basic Research and the Japan Society for Promotion of Science is No. 21-54-50001 and JPJSBP120214813.

The authors apologize for this error and state that this does not change the scientific conclusions of the article in any way. The original article has been updated.

## Publisher's Note

All claims expressed in this article are solely those of the authors and do not necessarily represent those of their affiliated organizations, or those of the publisher, the editors and the reviewers. Any product that may be evaluated in this article, or claim that may be made by its manufacturer, is not guaranteed or endorsed by the publisher.

