# Supplementary figures and images for: Serial Block-Face Scanning Electron Microscopy Reveals That Intercellular Nuclear Migration Occurs in Most Normal Tobacco Male Meiocytes
Source: Front Plant Sci. 2021 May 7;12:672642. doi: 10.3389/fpls.2021.672642 (PMC8138938; doi:10.3389/fpls.2021.672642)

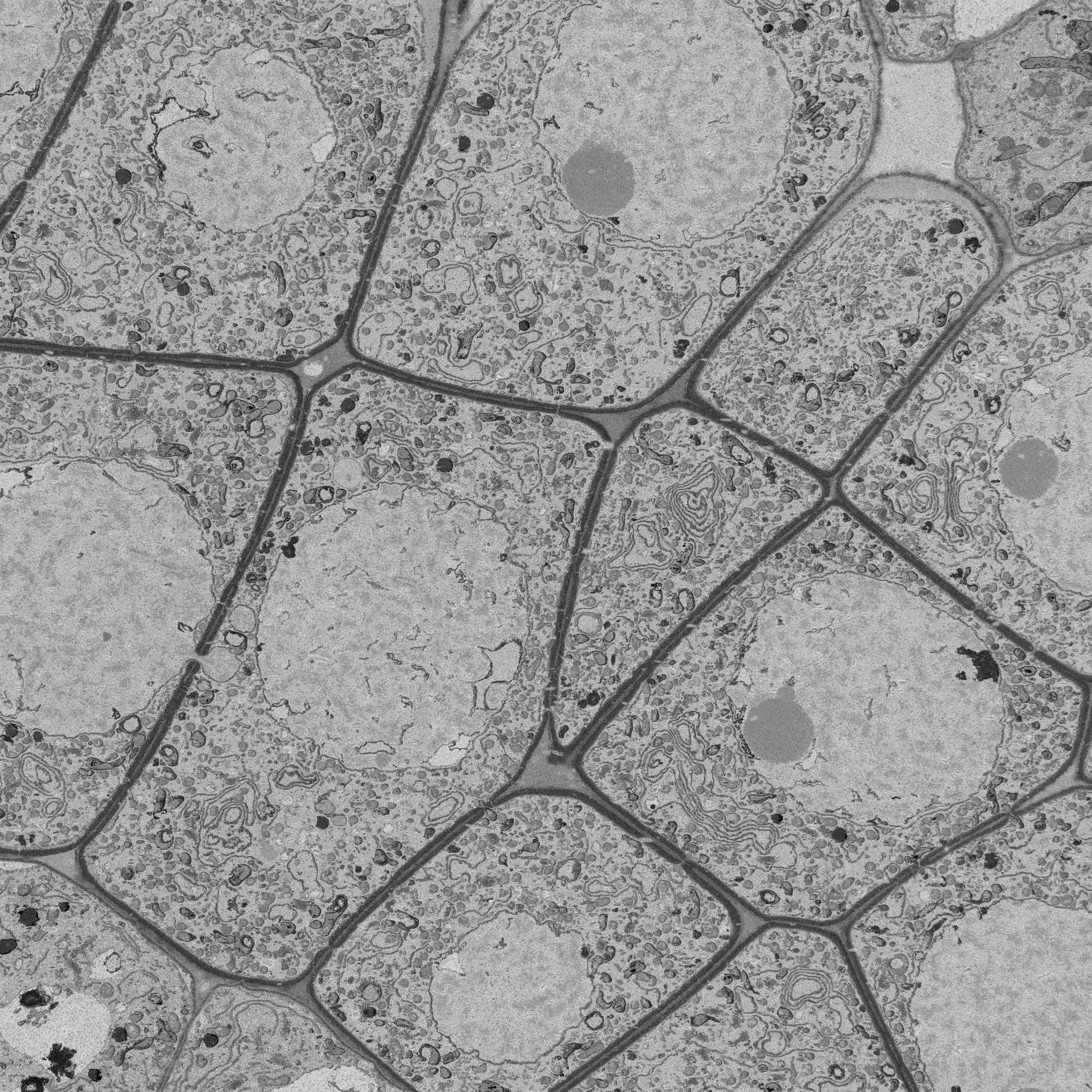

Supplement: Supplementary Figure 1 — Tobacco meiocytes at leptotene (original unlabeled image from Figure 2A). [file Image_1.TIF]

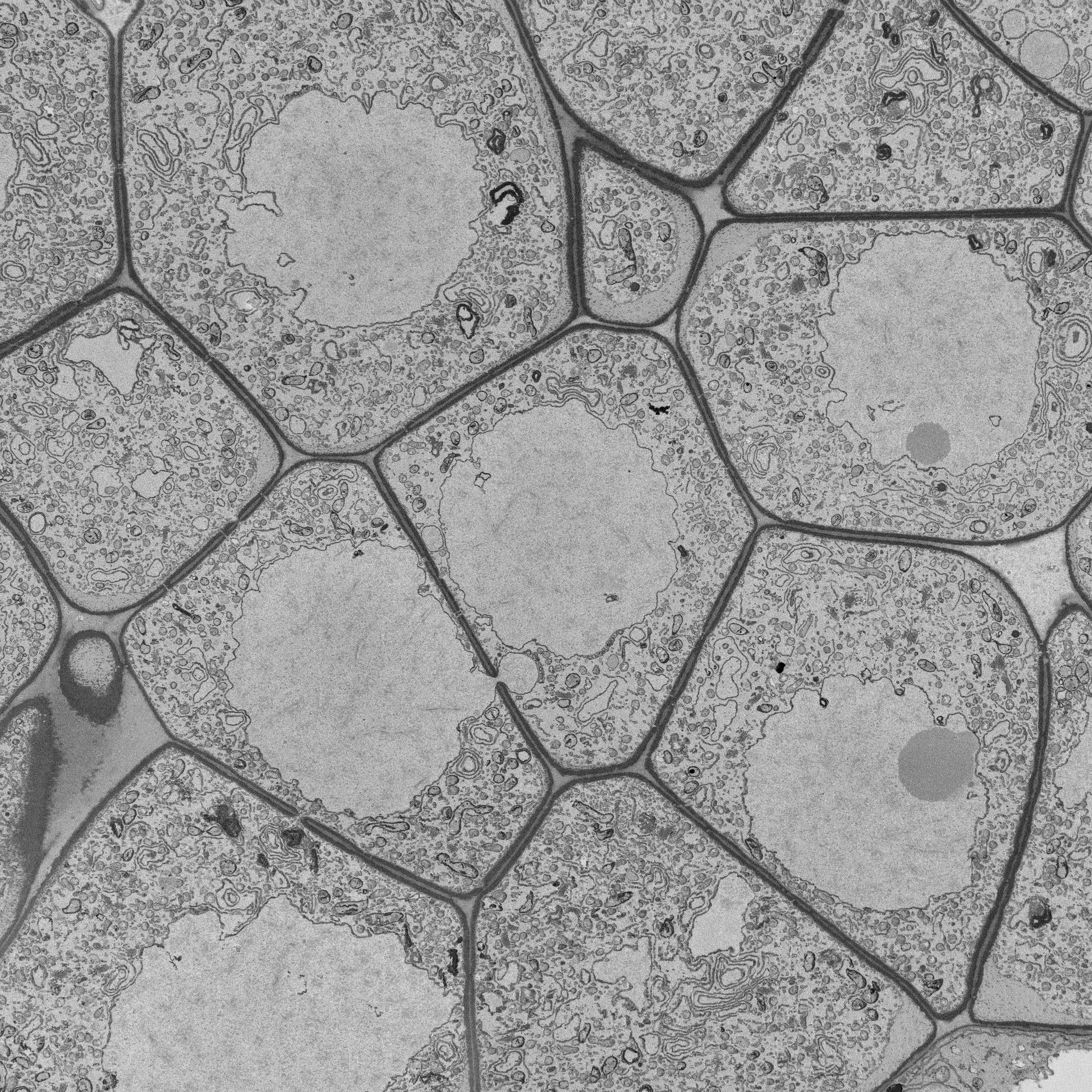

Supplement: Supplementary Figure 2 — Tobacco meiocytes at zygotene (original unlabeled image from Figure 2A). [file Image_2.TIF]

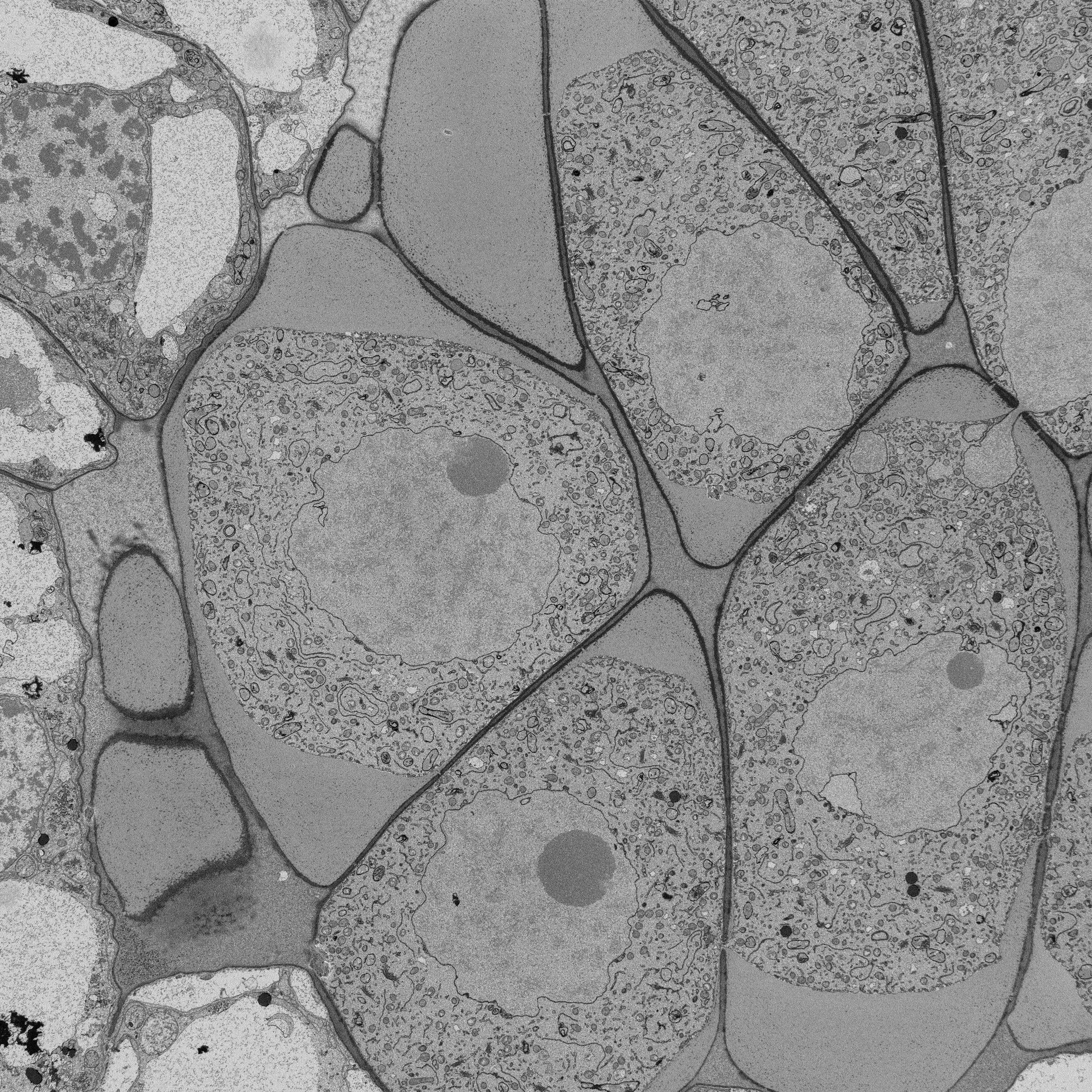

Supplement: Supplementary Figure 3 — Tobacco meiocytes at pachytene (original unlabeled image from Figure 2A). [file Image_3.TIF]

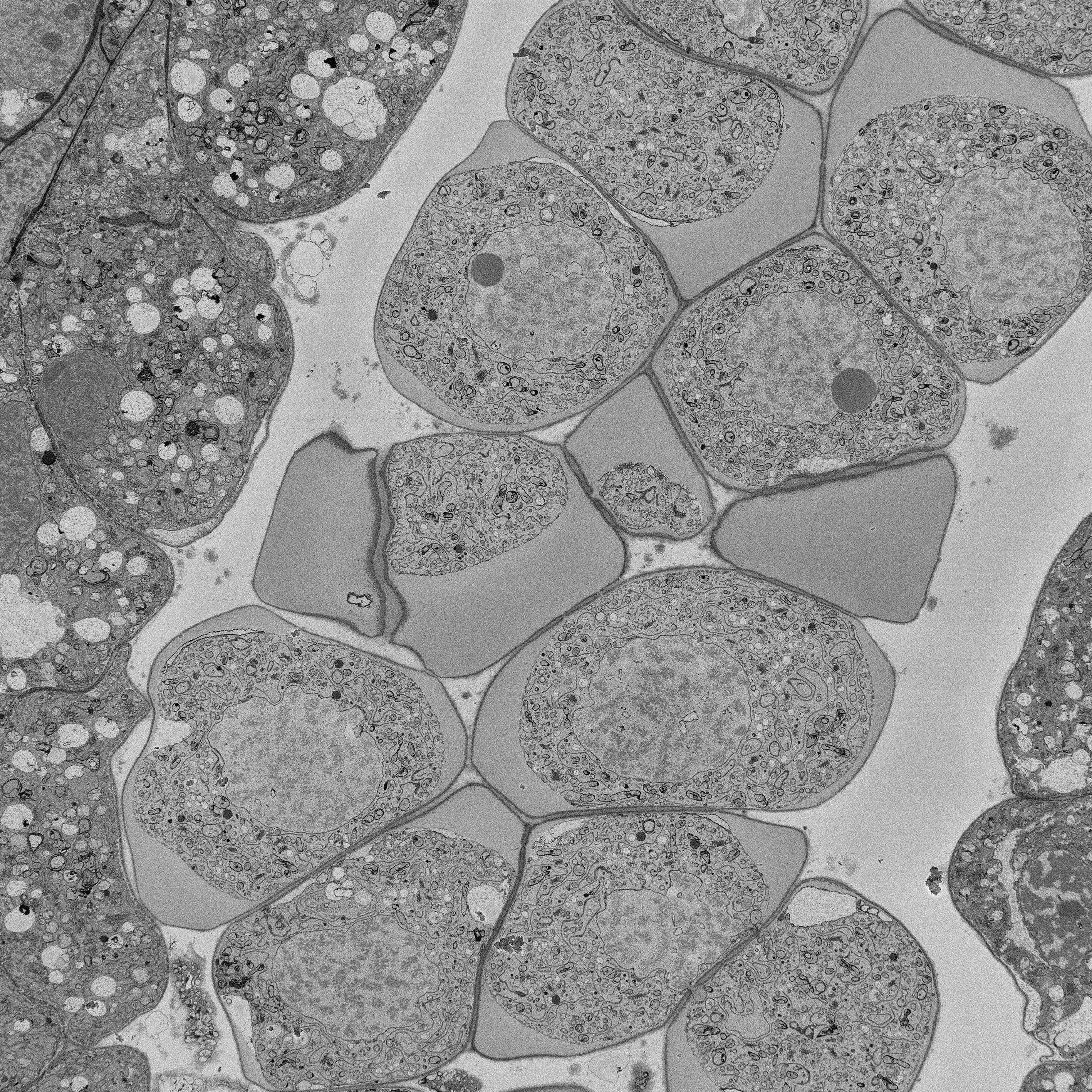

Supplement: Supplementary Figure 4 — Tobacco meiocytes at diplotene (original unlabeled image from Figure 2A). [file Image_4.TIF]

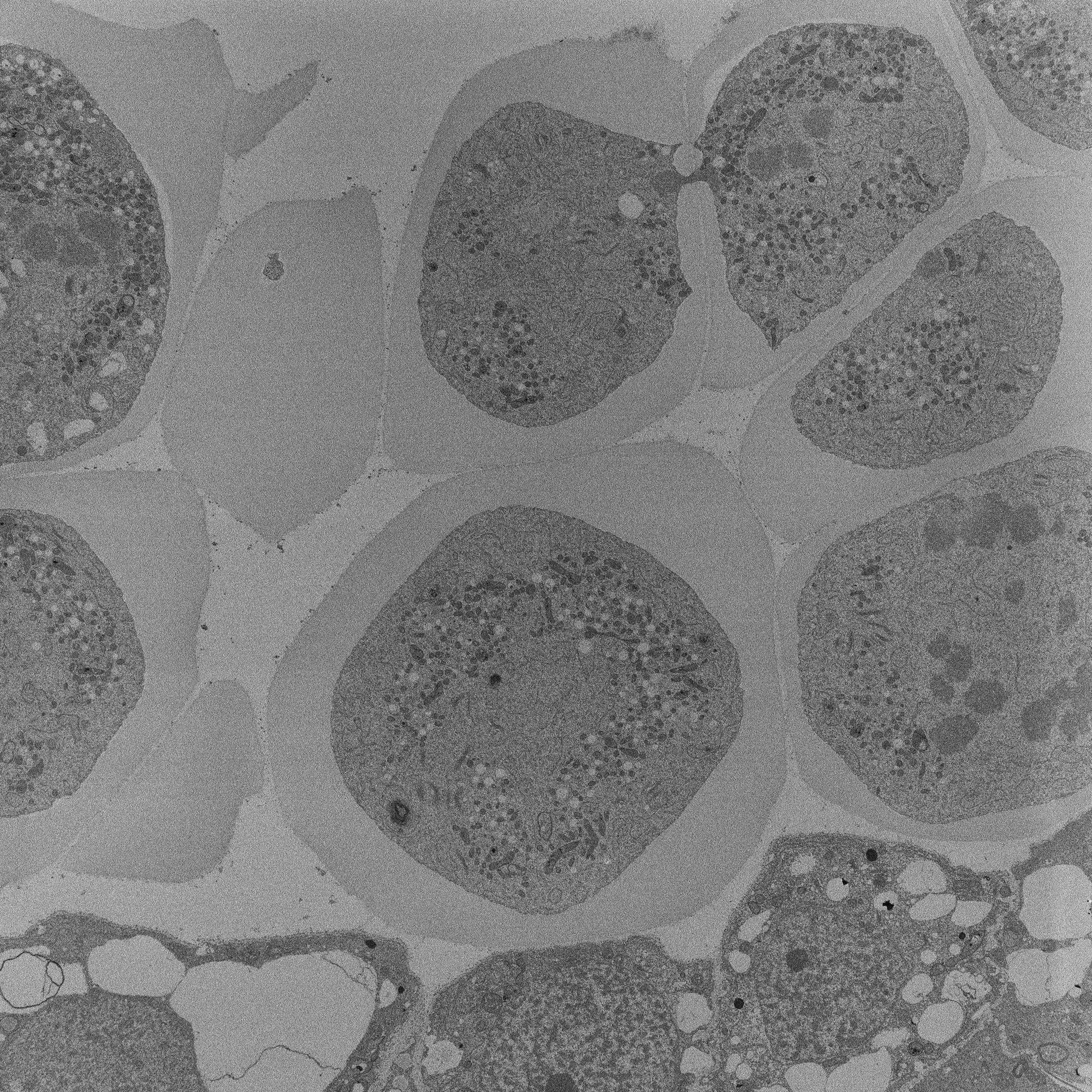

Supplement: Supplementary Figure 5 — Tobacco meiocytes at anaphase I (original unlabeled image from Figure 2A). [file Image_5.TIF]
